# Intravascular Imaging Guidance for Percutaneous Coronary Interventions

**DOI:** 10.3390/jcm14227994

**Published:** 2025-11-11

**Authors:** Marco Spagnolo, Daniele Giacoppo, Antonio Greco, Davide Capodanno

**Affiliations:** Division of Cardiology, Azienda Ospedaliero-Universitaria Policlinico “G. Rodolico—San Marco”, University of Catania, 95123 Catania, Italy; marcospagnolo93@gmail.com (M.S.); a.greco90@gmail.com (A.G.); davide.capodanno@unict.it (D.C.)

**Keywords:** intravascular imaging, intravascular ultrasound, optical coherence tomography, near-infrared spectroscopy, coronary artery disease, vulnerable plaque, percutaneous coronary intervention, drug-eluting stent

## Abstract

Intravascular imaging (IVI), particularly intravascular ultrasound (IVUS) and optical coherence tomography (OCT), addresses the intrinsic limitations of two-dimensional coronary angiography by offering high-resolution information regarding vessel and plaque morphology before percutaneous coronary intervention (PCI) as well as enabling accurate assessment of stent expansion and apposition after implantation. These anatomical insights can translate into improved procedural success and late clinical outcomes. The magnitude of benefit appears closely related to lesion morphology and procedural complexity. While angiographic guidance may be sufficient in straightforward anatomies, IVI assumes a pivotal role in complex disease subsets. IVUS, with its deeper tissue penetration, real-time imaging capability, and lack of need for contrast flushing, is particularly advantageous for large-vessel interventions, chronic total occlusions, and contrast-sparing strategies. In contrast, OCT, offering superior axial resolution, excels in characterizing plaque composition and in detecting stent-related complications. Hybrid IVUS-OCT catheters have the potential to integrate the complementary strengths of both IVI modalities, thereby streamlining procedural workflows and broadening clinical applicability. Although current guidelines endorse IVI use in anatomically complex coronary artery disease, real-world adoption remains low, largely influenced by operator proficiency, regional differences, and reimbursement arrangements. Further research is warranted to identify lesion subsets in which one modality confers clear clinical benefit and to delineate the threshold of procedural complexity at which IVI becomes cost-effective.

## 1. Introduction

Coronary angiography is the primary invasive procedure used for diagnosing coronary artery disease and guiding percutaneous coronary intervention (PCI) [1]. However, it has inherent limitations in accurately assessing complex vascular anatomies and plaque morphology as it provides low-resolution, two-dimensional images and evaluates the vessel wall indirectly through contrast media filling, without information on plaque composition [1]. To address these limitations, intravascular imaging (IVI) technologies have been developed, offering detailed luminal assessment and enabling proper characterization of atherosclerotic plaque features [2].

Advances in the understanding of vascular biology and atherosclerotic plaque progression, alongside the growing complexity of PCI procedures and the introduction of novel devices, have further underscored the diagnostic and prognostic value of IVI [3,4,5,6,7,8,9]. Accordingly, over the past decades, substantial improvements in IVI device performance, primarily reduced catheter profile and enhanced crossability, and image resolution have been implemented in clinical practice to maximize clinical usefulness and broaden the adoption [10,11]. To date, multiple randomized clinical trials and meta-analyses support the use of IVI to guide and optimize PCI, establishing the basis for strong recommendations in American and European guidelines for intravascular ultrasound (IVUS) and optical coherence tomography (OCT) in anatomically complex lesions [12,13,14,15,16,17].

Consequently, IVI is now regarded as the reference standard for guiding complex PCI and optimizing procedural outcomes, with an emerging role in lesion-specific risk stratification to support tailored therapeutic strategies [12,13,18]. IVI guidance is pivotal for accurately measuring plaque extension and luminal obstruction, assessing stent expansion and apposition, and detecting early suboptimal PCI results, at the cost of a negligible or absent risk of acute vascular complications, arrhythmias, periprocedural myocardial infarction, and acute kidney injury [19,20,21]. In parallel, IVI is also an established in vivo gold standard for identifying vulnerable plaque features amenable to aggressive stabilizing medical therapy or preventive interventions, and for anticipating procedural requirements that could reduce the risk of adverse cardiac events [22,23,24]. Moreover, IVI guidance to ensure optimal procedural results and to assess intermediate non-culprit lesions may be particularly advantageous in patients who underwent complex interventions and are at high thrombotic risk, especially when high bleeding risk conditions require shorter and less intensive antiplatelet therapies than usually recommended, or when early discontinuation of antiplatelet therapy is more likely [25,26,27,28,29,30,31]. Although still under investigation, IVI also shows promise in guiding targeted lipid-lowering and anti-inflammatory pharmacotherapy that could alter the natural history of coronary artery disease in some plaque and patient phenotypes [32,33,34,35,36,37].

Despite these advantages, IVI adoption remains highly variable across regions, ranging from approximately 5–15% in Europe and the United States, where use remains selective, to 75–85% in Japan, where routine application is standard [38,39,40]. This disparity is influenced by multiple factors, including limited familiarity with catheters and consoles, gaps in proper image interpretation, concerns about increased procedural times and workflow disruption, and misperceptions of recent clinical trial evidence. However, reimbursement policy remains a primary determinant of this variability, shaping institutional and national IVI adoption patterns [41].

In this review, we provide an overview of currently available IVI techniques, examine current recommendations and supporting evidence for their use in PCI guidance, covering procedural planning, stenting optimization, and early results assessment, and discuss future directions in the field.

## 2. Overview of Intravascular Imaging Modalities

A variety of invasive and non-invasive imaging modalities are available to characterize coronary vessel morphology, quantify plaque burden, and assess plaque distribution and composition. These techniques provide detailed evaluation regarding specific aspects of target lesions and can guide PCI across different patterns of coronary artery disease with specific advantages and limitations [42,43,44,45].

IVUS and OCT provide real-time, high-fidelity imaging that enables morphological assessment of both plaque and vessel architecture, as well as procedural optimization by optimal stent expansion and apposition [42,43,44,45,46]. Nevertheless, although OCT has a higher resolution than IVUS, data indicating improved long-term hard clinical outcomes after procedural guidance and post-intervention evaluation with IVUS seem to be more uniform than those obtained with OCT based on the consistency of results across trials and different settings [11,17,47]. In addition, information provided by IVUS, OCT, and near-infrared spectroscopy (NIRS) has shown high correlation with histology and has been extensively employed for the assessment of plaque characteristics [24,48,49,50,51,52,53]. Yet, since these modalities carry a risk of vessel injury during image acquisition and, more in general, procedure-related complications, they are unsuitable for systematic screening of patients, especially in those without an overt indication for coronary angiography [54]. Moreover, depending on anatomic factors, these catheter-based techniques may be incapable of inspecting distal segments and secondary coronary branches [55].

As a result, substantial efforts have also been directed toward the development of non-invasive imaging modalities, primarily coronary computed tomography angiography (CCTA), which offer the potential for broader patient screening, early identification of individuals requiring invasive assessment, guidance in treatment decision-making, and procedural planning for PCI [56,57]. Other emerging non-invasive modalities, such as magnetic resonance imaging (MRI) and positron emission tomography (PET), show promise but remain under development [58,59]. Nevertheless, non-invasive techniques offer lower spatial resolution and limited specificity compared to IVI for detailed assessment of plaque characteristics. Their utility is inherently constrained in the setting of acute coronary syndromes (ACS) and they are unsuitable for immediate post-PCI evaluation.

### 2.1. Intravascular Ultrasound

Gray-scale IVUS uses either mechanical rotating transducers or solid-state electronic phased-array transducers mounted at the tip of the imaging catheter, which is positioned across the coronary segment of interest [55]. These transducers emit ultrasound waves and generate cross-sectional images by converting the acoustic signals backscattered from the coronary structures [55]. Gray-scale IVUS enables the definition of segmental plaque burden and geometry, provides precise measurements of the degree of luminal obstruction, identifies thrombotic material, and guides PCI [55]. However, the qualitative assessment of plaque composition based on tissue echogenicity (soft: lower echogenicity than adventitia; fibrous: intermediate echogenicity; calcified: higher echogenicity than adventitia with acoustic shadowing; mixed: multiple acoustic signals) requires post-processing through validated algorithms, such as integrated-backscatter IVUS (IB-IVUS), iMAP, and virtual histology IVUS (VH-IVUS), to significantly improve plaque characterization accuracy and reduce operator-dependent variability [48,55,60,61,62,63,64]. IB-IVUS uses time-domain information directly extracted from radiofrequency signals, iMAP applies a spectral pattern recognition algorithm based on fast Fourier transformation, and VH-IVUS, the most widely used technique for plaque characterization by IVUS, relies on spectral analysis of the frequency and amplitude of ultrasound signals [48,61,62,63,64]. All these post-processing techniques overlay a color-coded tissue map onto grayscale images to depict plaque composition [48,61,62,63,64]. The VH-IVUS algorithm assigns colors as follows: fibrous tissue (dark green), fibro-fatty tissue (light green), necrotic core (red), and dense calcium (white). VH-IVUS has been validated against histology in autopsy specimens, demonstrating diagnostic accuracies of 79.7–87.1% for fibrous tissue, 81.2–87.1% for fibrofatty tissue, 85.5–88.3% for necrotic cores, and 92.8–96.5% for calcium [48,49,50,61,62].

### 2.2. Optical Coherence Tomography

Frequency-domain OCT catheters emit near-infrared light through a rotating single optical fiber coupled with an imaging lens [43,65,66,67]. The interference pattern at different wavelengths (~1.25–1.35 µm) is processed by Fourier transformation to provide the amplitude profile and echo time delay of the light backscattered off the coronary structures [43,65,66,67]. Fibrotic tissue produces a relatively homogeneous highly backscattering signal, calcification determines a signal-poor area with sharply delineated borders, and lipid pool is associated with signal-poor areas with vaguely defined borders.

OCT has the highest resolution among the contemporary coronary imaging modalities [43,65,66,67]. In particular, an axial resolution (10–15 µm) about ~10 times greater than that of IVUS enables the assessment of superficial plaque composition and microstructures [43,65,66,67]. Histopathology studies have shown that OCT differentiates lipid from fibrous tissue accurately (sensitivity 90–94%, specificity 90–92%) and enables the direct measurement of cap thickness and the quantification of calcification compared with IVUS [63,67,68,69,70]. In addition, OCT properly identifies plaque erosion and rupture, red and white thrombi, microchannels, cholesterol crystals, neovascularization, and in some circumstances macrophage infiltration at the border of the necrotic core and fibrous cap (punctate signal-rich clusters) [63,67,71,72].

### 2.3. Near-Infrared Spectroscopy

Near-infrared spectroscopy (NIRS), an established method in physical sciences to characterize the chemical composition of various biomaterials, was introduced as a catheter-based system to complement IVUS information and overcome its inherent technical limitations in detecting some vulnerable plaque characteristics [73,74,75,76,77]. NIRS relies on the spectroscopic analysis of the light absorbed and backscattered off the tissues to provide a color-coded representation (chemogram) of the arterial wall cholesterol levels [73,74,75,76,77]. Sensitivity and specificity are 90% and 93% for the lipid pool, 77% and 93% for the thin cap, and 84% and 91% for the presence of inflammation [74]. Thus, NIRS overtakes IVUS and OCT in detecting lipid-rich plaques and discriminating between calcium and necrotic core [75,76]. The primary compositional measure of NIRS is the lipid-core burden index (LCBI) which quantifies the lipid content, calculated by determining the proportion of yellow pixels relative to the total number of pixels multiplied by 1000 [77]. The maxLCBI_4mm_, ranging from 0 to 1000, quantifies the maximal extent of lipid-rich plaques, expressed as LCBI, within targeted areas segmented into 4 mm sections [77]. Importantly, while NIRS cannot guide stent optimization or assess acute procedural results, it complements IVUS or OCT by providing robust lipid quantification to identify plaques prone to rupture that may benefit from intensive lipid-lowering therapy and preventive PCI.

### 2.4. Intravascular Ultrasound and Optical Coherence Tomography for Guiding Percutaneous Coronary Interventions

**Figure 1:**
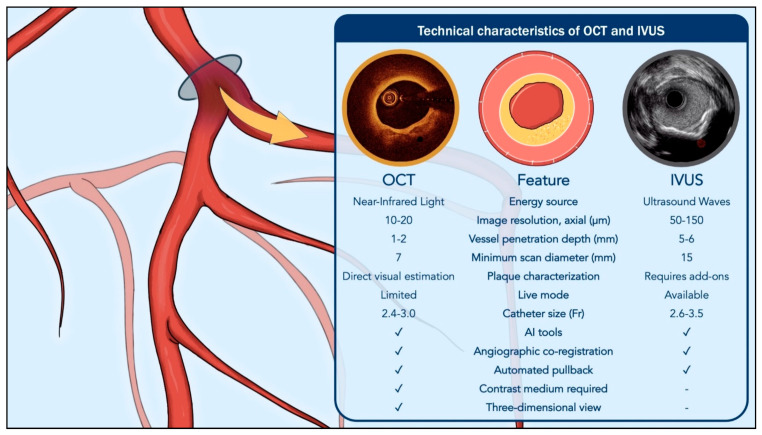
Comparative technical characteristics of optical coherence tomography and intravascular ultrasound. Abbreviations: AI, Artificial Intelligence; IVUS, Intravascular Ultrasound; OCT, Optical Coherence Tomography.

**Figure 2:**
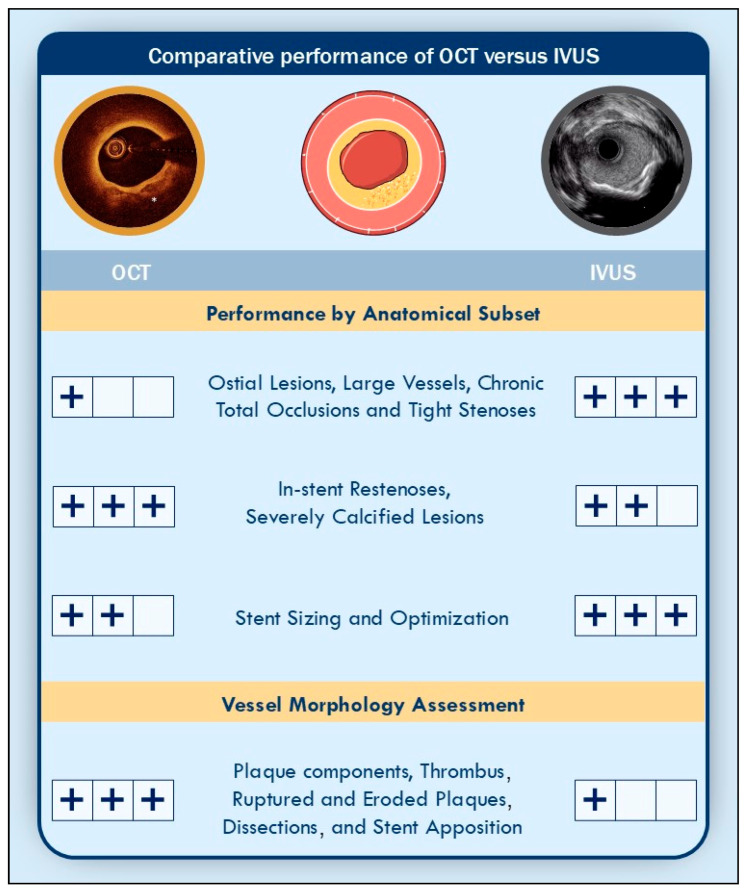
Performance of optical coherence tomography versus intravascular ultrasound by anatomical subset. Abbreviations: IVUS, Intravascular Ultrasound; OCT, Optical Coherence Tomography.

Key technical differences and expected performance characteristics of the two modalities are summarized in Figure 1 and Figure 2.

#### 2.4.1. Procedural Planning

In the context of procedural planning, the high axial resolution of OCT enables distinct advantages in the assessment of endoluminal material, vessel surface, superficial arterial wall and plaque tissue layers, especially when there is no significant amount of red thrombus, lipid, or necrotic core attenuating the light signal [47,78]. These properties enable quantification of the thrombotic burden upon ruptured or eroded plaques responsible for ACS and, when this event has not yet occurred, allow assessment of plaques at high risk of rupture [67]. This makes OCT the method of choice for measuring cap thickness, a key marker of plaque vulnerability, with an increased risk of rupture or erosion when it is <65 μm [47,79]. In contrast, although contemporary high-definition systems have reduced the resolution gap with OCT, IVUS generally infers the presence of a thin-cap fibroatheroma from the absence of fibrous tissue between the lumen and the plaque, as determined by tissue echogenicity post-processing [24,55,80,81]. OCT also generally allows accurate quantification and spatial mapping of calcified tissue [66]. In contrast, IVUS assessment of heavily calcified lesions is often limited by acoustic shadowing resulting from strong ultrasound reflection, which can obscure deeper vessel structures [47]. Moderate or severe calcified lesions on IVI frequently require more aggressive lesion preparation with non-compliant and/or cutting/scoring balloons [82,83]. Severe calcifications may also necessitate adjunctive debulking devices, such as rotational/orbital atherectomy, intravascular lithotripsy, and laser before stenting [82,83].

In the context of procedural planning, however, it is also important to consider that OCT light has limited penetration depth (1.0–2.5 mm), approximately four times lower than that of IVUS (4.0–8.0 mm), and exhibits reduced diffusion through lipid-rich plaques and necrotic cores [11]. These limitations can sometimes compromise the accuracy of OCT in assessing plaque depth and extent, making IVUS generally superior for measuring the atheroma burden and displaying the entire vessel [43,55]. For the same reasons, IVUS is generally more reliable than OCT for measuring reference vessel diameter and guiding stent size selection, particularly in large vessels. Nevertheless, both IVUS and OCT improve device size selection compared with angiography through measurements of the reference vessel diameter based on external elastic lamina at the proximal, distal, and lesion sites [44,84,85]. These measurements, in particular the distal site estimate, are generally rounded down by approximately 0.25 mm to the nearest stent size [84,85]. Diffuse long lesions, which result in tapered reference vessel diameter across the target segment, require that both proximal and distal mean external elastic lamina diameter measurements be jointly considered. In these circumstances, stent size should generally be rounded down, with subsequent proximal stent overexpansion using non-compliant balloons [84,85]. In cases where the external elastic lamina is not sufficiently visible, a lumen-based sizing is recommended and the corresponding stent diameter should be rounded up by at least 0.25–0.5 mm, depending on the amount of plaque burden [84,85].

Both IVUS and OCT supersede coronary angiography in accurately defining lesion length. The importance of proper stent length selection is underscored by the consistent identification of incomplete lesion coverage as one of the predictors of target lesion failure (TLF) and major adverse cardiac events (MACE) [43,44,54]. It is advised to avoid selecting a landing zone in areas of thin-cap fibroatheroma, lipid-rich plaque, and eccentric calcium, due to the increased risk of edge dissections and increased risk of peri-procedural myocardial infarction after soft plaque tissue embolism [44,85,86].

#### 2.4.2. Percutaneous Coronary Intervention Optimization

Regarding PCI optimization, sufficient stent expansion is instrumental, as stent underexpansion is strongly correlated with target lesion-related adverse cardiac events [44,87,88,89]. After ILUMIEN III and IV, a minimum stent area (MSA) achieved at the proximal and distal segments ≥90% and ≥95% of the corresponding reference lumen areas are considered indicative of acceptable and optimal stent expansion, respectively [84,85,90,91]. However, in previous studies, an MSA >90% or event >80% of the average reference external elastic lamina was considered indicative of acceptable stent expansion [44,85,91,92]. In addition, some studies defined acceptable stent expansion as an absolute MSA >4.5 mm^2^ or >5.5 mm^2^ for non-left main coronary artery segments, and MSAs >8.0 mm^2^ and >7.0 mm^2^, for proximal and distal left main coronary artery segments, respectively [44,93,94]. In the DOCTOR trial, an OCT-defined absolute MSA of 5.44 mm^2^ in non-left main coronary artery segments was the optimal cut-off to predict postprocedural fractional flow reserve >0.90 [92].

Stent malapposition, defined as the complete separation between the stent struts and the endothelial surface, can occur immediately after stent deployment (acute malapposition) or develop later (late malapposition) [84,85]. OCT is generally more sensitive than IVUS in detecting malapposition due to higher resolution [93,95]. Although the clinical relevance of acute malapposition remains debated and some studies suggest that when it occurs without stent underexpansion, it is generally not linked to higher rates of stent failure, certain scenarios warrant correction, including proximal edge malapposition that may hinder re-wiring, extensive malapposition over long segments (>3 mm), or malapposition accompanied by underexpansion [95,96,97,98,99,100,101,102].

In the context of early complication detection, OCT provides superior capability for detecting edge dissections compared with IVUS [11,44,85,91]. After stenting, OCT provides high-resolution information leading to the frequent identification of edge dissections that are not detectable by angiography in approximately 80% of cases [85,91]. However, most of these dissections are usually minor and have no clinical and prognostic impact. Evidence from previous studies defined some cut-off values of tissue rim width, dissection length, intramural hematoma arc grades, and cavity depth to identify those lesions requiring treatment to avoid adverse clinical outcomes [85,91,93]. In addition, the distal edge location seems to have a stronger association with adverse cardiac events [85,91,93].

#### 2.4.3. Technical Considerations

Since red blood cells scatter near-infrared light, OCT image acquisition requires contrast media injection to transiently displace blood during high-speed motorized pullback. This requirement limits the feasibility of real-time imaging, which can be particularly valuable during PCI for chronic total occlusions [11,67,79]. In addition, time-demanding, complex interventions requiring repeated IVI support pose issues of multiple contrast injections with OCT, though contrast-sparing techniques, such as targeted pullback activation with low-volume injections (3–5 mL) via guide extension catheters, have been introduced [103,104]. It is also worth noting that ostial and otherwise challenging anatomies that impair optimal visualization and displacement of blood may limit the usefulness of OCT [43,65,66,67].

OCT is less prone than certain mechanical IVUS systems to rotational artifacts and provides enhanced visualization of side branches and guidewire positioning [11]. These attributes make OCT particularly advantageous for assessing bifurcation lesions and for the meticulous optimization of stent deployment and apposition [11]. Nevertheless, image acquisition is not possible during wire manipulation, and several pull-backs during injection of contrast media are required in the context of complex bifurcation disease.

Given that drug-coated balloon use is growing for the treatment of specific patterns of coronary artery disease and coronary dissection is frequently observed after drug-coated balloon angioplasty, the use of IVI has been considered to predict those lesions that may progress into major, flow-limiting dissections, and ultimately periprocedural myocardial infarction and abrupt vessel closure [8,105,106,107,108]. However, in the absence of validated criteria, the use of IVI after drug-coated balloon angioplasty, especially OCT, may produce overtreatment and unnecessary bailout stenting when it is established that angiographically defined minor dissections unfrequently progress and cause adverse cardiac events [8,105,106,107,108].

In aggregate, in straightforward anatomies, where lesion complexity is modest and patient comorbidity minimal, either OCT or IVUS supplies adequate intravascular detail beyond angiography alone. In more challenging settings, however, their inherent strengths and limitations translate into differential performance; optimal imaging therefore hinges on matching lesion-specific demands to modality capabilities and, equally, to the operator’s technical proficiency.

## 3. Guidelines Recommendations

Current American and European guideline recommendations for the use of IVI in PCI are summarized in Table 1. The integration of IVI, specifically IVUS and OCT, into routine PCI practice has been increasingly endorsed by contemporary major cardiovascular society guidelines, particularly but not exclusively for anatomically complex coronary lesions. Current recommendations from both American and European societies are grounded in the high-level evidence demonstrating that IVI improves technical outcomes and translates into a reduction in long-term adverse cardiac events [17,109].

For patients with stable coronary artery disease undergoing PCI of anatomically complex lesions, including left main stem, long lesions, and bifurcations with significant disease of the side branch, the 2024 European Society of Cardiology (ESC)/European Association for Percutaneous Cardiovascular Interventions (EAPCI) Guidelines on Chronic Coronary Syndromes provide a Class I, Level of Evidence A recommendation for the use of IVUS or OCT [12]. This position is supported by recent randomized controlled trials and a comprehensive network meta-analysis demonstrating that improved procedural precision with IVI translates into lower rates of TLF [17,109]. In contrast, the 2023 American College of Cardiology (ACC)/American Heart Association (AHA)/Society for Cardiovascular Angiography and Interventions (SCAI) Guidelines for Chronic Coronary Artery Disease does not provide a specific recommendation regarding IVI use, possibly reflecting the fact that this topic was already addressed in prior myocardial revascularization guidelines issued by the same societies [110].

In the setting of ACS, the 2023 ESC/EAPCI Guidelines recommend that the use of IVI should be considered to guide PCI (Class IIa, Level of Evidence A). Additionally, OCT may be considered in cases of angiographically ambiguous culprit lesions (Class IIb, Level of Evidence C), reflecting its superior axial resolution and ability to resolve fine structural details relevant for plaque characterization [111]. More recently, the 2025 ACC/AHA/SCAI Guideline for the Management of ACS has elevated the recommendation for IVI to the highest level, assigning a Class I, Level of Evidence A endorsement for IVUS or OCT guidance during PCI in ACS patients with left main or other complex lesions to reduce ischemic events [16].

## 4. Evidence Supporting Intravascular Imaging Guidance for Percutaneous Coronary Intervention

### 4.1. Intravascular Ultrasound Versus Angiography

Key characteristics of randomized clinical trials comparing IVUS with angiography are illustrated in Table 2. Early randomized investigations of IVUS established superior angiographic outcomes (i.e., late-lumen loss, minimal-lumen diameter and post-procedural fractional flow reserve) compared with angiographic guidance alone [92,112,113]. Subsequent larger studies, powered for long-term clinical outcomes, confirmed that these procedural advantages translated into prognostic improvements [92,112,113]. The IVUS-XPL trial, including 1400 patients with lesions requiring implantation of stents ≥28 mm in length, demonstrated the superiority of IVUS guidance over angiography in reducing one-year MACE, defined as cardiac death, target lesion-related myocardial infarction, or ischemia-driven target lesion revascularization (2.9% vs. 5.8%, respectively; hazard ratio [HR] 0.48; 95% confidence interval [CI] 0.28 to 0.83; *p =* 0.007) [114]. The benefit was primarily driven by a reduction in ischemia-driven target lesion revascularization, while differences in cardiac death and myocardial infarction were not statistically significant [114]. The long-term benefit of IVUS was confirmed at five-year follow-up, with MACE occurring in 5.6% of IVUS-guided cases compared with 10.7% in the angiography group [115]. Later, the ULTIMATE trial randomized 1448 patients, 55% of whom had multivessel disease, 31% had diabetes, and 25% had bifurcation lesions, to either IVUS- or angiography-guided PCI [87]. Target vessel failure (TVF), defined as cardiac death, target-vessel myocardial infarction, and clinically driven target-vessel revascularization, occurred in 2.9% of the IVUS group versus 5.4% in the angiography group (HR 0.53; 95% CI 0.31 to 0.90; *p =* 0.019) [87]. At three-year follow-up, IVUS remained superior to angiography alone, with TVF rates of 6.6% versus 10.7%, and a significant reduction in definite or probable stent thrombosis (0.1% vs. 1.1%; *p =* 0.020) [116]. In contrast, the GUIDE-DES trial randomized 1528 patients at nine East Asian centers to IVUS- or quantitative coronary angiography-guided PCI with the objective of demonstrating non-inferiority of angiography in terms of TLF, defined as a composite endpoint of cardiac death, target vessel myocardial infarction, and ischemia-driven target lesion revascularization [117]. At one year, TLF occurred in 3.8% of both groups (HR 1.00; 95% CI 0.60 to 1.68; *p =* 0.999), resulting in the non-inferiority of quantitative coronary angiography to IVUS in guiding PCI [117]. Recently, the large-scale IVUS-ACS trial enrolled 3505 patients with ACS, showing a significantly lower one-year TVF rate, defined as a composite of cardiac death, target vessel myocardial infarction, or clinically driven target vessel revascularization, associated with IVUS guidance compared with angiography guidance (4.0% vs. 7.3%; HR 0.55; 95% CI 0.41 to 0.74; *p* < 0.001), primarily driven by reductions in target vessel myocardial infarction and revascularization [118].

### 4.2. Optical Coherence Tomography Versus Angiography

Key characteristics of randomized clinical trials comparing IVUS with angiography are illustrated in Table 3. ILUMIEN III focused on the difference in early postprocedural outcomes among OCT, angiography, and IVUS [84]. Although OCT was associated with non-inferior MSA compared to IVUS, ILUMIEN III failed to demonstrate superiority of OCT over both angiography and IVUS [84]. One-year clinical outcomes did not reveal significant clinical differences between strategies, but the trial was not powered for clinical outcomes [84]. Later, the CALIPSO trial, including 143 patients with moderate-to-severe calcification, showed that OCT achieved significantly larger MSA compared to angiography [126]. Subsequently, the OCTOBER trial randomized 1201 patients with true bifurcation disease and reported a 2-year MACE (cardiac death, target-lesion myocardial infarction, or ischemia-driven target-lesion revascularization) incidence of 10.1% in the OCT-guided PCI group versus 14.1% in the angiography-guided PCI group (HR 0.70; 95% CI 0.50 to 0.98; *p =* 0.035), largely driven by reductions in target-vessel myocardial infarction and revascularization [127]. Approximately one third of patients did not undergo a two-stent strategy, showing an unexpectedly larger numerical benefit in 2-year MACE compared with those treated with a two-stent strategy [127]. More recently, the OCCUPI trial enrolling 1604 patients with multiple types of complex coronary artery disease (chronic total occlusions, long lesions, calcified plaques, bifurcations, unprotected left main disease, small vessels, intracoronary thrombus, stent thrombosis, in-stent restenosis, and bypass graft lesions) demonstrated a significant reduction in 12-month MACE (composite of cardiac death, myocardial infarction, stent thrombosis, or ischaemia-driven target-vessel revascularization) in the OCT-guided PCI group compared with the angiography-guided PCI group (5.0% vs. 7.0%; HR 0.62; 95% CI 0.41 to 0.93; *p =* 0.023) [128]. Of note, stroke, bleeding, and contrast-induced kidney injury rates were similar between groups [128]. In contrast, ILUMIEN IV, the largest available trial on OCT- versus angiography-guided PCI, showed no significant difference in two-year TVF (composite of death from cardiac causes, target-vessel myocardial infarction, or ischemia-driven target-vessel revascularization) between treatment groups (7.4% vs. 8.2%; HR 0.90; 95% CI 0.67–1.19; *p =* 0.450), despite patients assigned to OCT achieving larger postprocedural MSA compared with those assigned to angiography, in line with the previous trials [90]. The interpretation of these findings is challenging and likely multifactorial. In this regard, it should be noted that ILUMIEN IV predominantly enrolled patients with single-vessel coronary artery disease, and the proportion of some subsets of lesions previously associated with improved outcomes after intravascular imaging was lower than in other trials. In addition, some criteria of clinical and anatomical complexity may have been overestimated when designing the trial. In any case, the heterogeneous results observed across trials underscore that some patterns of coronary artery disease and operator’s expertise may play a pivotal role. Taken together, these findings reinforce the principle that the clinical benefits of OCT-guided PCI are most pronounced in anatomically or procedurally complex scenarios, where lesion morphology, vessel size, or architecture challenge the interpretative capacity of angiographic imaging, and less evident in low-risk, single-vessel disease, where imaging may offer procedural refinements without clear impact on hard clinical endpoints.

### 4.3. Intravascular Ultrasound Versus Optical Coherence Tomography

**Figure 3:**
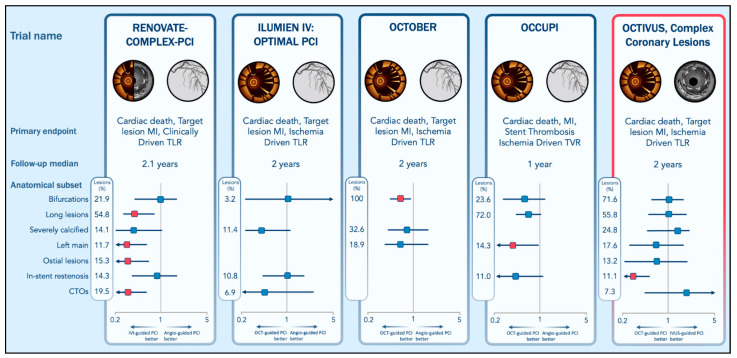
Impact of IVI across anatomical lesion subsets. Abbreviations: CTO, Chronic Total Occlusions; IVI, Intravascular Imaging; IVUS, Intravascular Ultrasound; MI, Myocardial Infarction; OCT, Optical Coherence Tomography; TLR, Target Lesion Revascularization. Red squares denote statistically significant effects. Blue squares denote non-statistically significant effects. Adapted with permission from Capodanno & Spagnolo [128].

Figure 3 summarizes the impact of IVI across various anatomical lesion subsets, highlighting where specific modalities may offer the greatest procedural and clinical advantage.

The figure illustrates the effect size of the primary endpoint across anatomical lesion subsets from four recent trials comparing IVI techniques for guidance of percutaneous coronary intervention. The prevalence of each anatomical lesion subset within each trial is reported. The definition of each anatomical subset is trial-defined and the results for each subgroup should be interpreted as hypothesis-generating. 

A limited number of randomized trials have directly compared IVUS and OCT. As previously mentioned, ILUMIEN III provided information on the comparison between IVUS and OCT. The study demonstrated non-inferiority of OCT relative to IVUS in terms of post-procedural MSA and was underpowered to assess clinical outcomes [84]. More recently, in the less explored setting of ACS, OPINION-ACS reported that immediately post-PCI, the MSA was numerically smaller with OCT compared to IVUS (*p =* 0.096), while the incidence of proximal edge dissections and irregular tissue protrusions was significantly lower in the OCT group [133]. At eight months, in-stent minimal luminal area measured 4.91 mm^2^ in the OCT-guided group and 4.76 mm^2^ in the IVUS-guided group, resulting in the non-inferiority of OCT guidance to IVUS guidance (*p* < 0.001) [133].

Against this background, two major randomized trials have directly compared OCT and IVUS for clinical outcomes, neither of which demonstrated substantial differences (Table 4) [134,135]. The largest of these trials, OCTIVUS, included 2008 patients and demonstrated the non-inferiority of OCT to IVUS with respect to a composite endpoint of cardiac death, target-vessel myocardial infarction, or ischemia-driven target-vessel revascularization at one year (2.5% vs. 3.1%, respectively; *p* < 0.001 for noninferiority). However, the trial was powered based on an anticipated 8.0% event rate in the IVUS group, whereas the observed event rate was less than half of this estimate. This substantial discrepancy rendered the trial underpowered and introduced bias in favor of a noninferiority conclusion [136]. Taken together, the available randomized data comparing OCT and IVUS include insufficient numbers of clinical events to definitively establish meaningful differences in patient outcomes.

One approach to discerning potential differences between IVI modalities is to examine populations anticipated to have higher rates of adverse clinical events, such as those with complex coronary lesions, where the specific advantages of each imaging technique may be more likely to manifest. A prespecified sub-analysis of the OCTIVUS trial offered valuable insight by evaluating outcomes in a cohort of 1475 patients with complex coronary anatomy, representing 73.5% of the total study population [139]. The lesions included in this analysis were predominantly bifurcations (~72%), with roughly half of the patients (~56%) presenting with long diffuse disease [139]. Patients undergoing OCT-guided PCI received, on average, an additional 40 mL of contrast dye during procedures, without any corresponding increase in contrast-induced acute kidney injury, and the PCI durations were approximately six minutes shorter [139]. OCT-guided PCI was associated with smaller mean MSA and reduced stent expansion and was less likely to meet stent-optimization criteria [139]. Although PCI-related complications were more frequent in the IVUS-guided group, none of these complications appeared to be directly attributable to the differences in size between OCT and IVUS imaging catheters [139]. At a median of two years, the Kaplan–Meier estimate of the primary composite endpoint was 6.5% for the OCT-guided group and 7.4% for the IVUS-guided group (HR 0.87; 95% CI 0.59 to 1.29; *p =* 0.50) [139]. This result remained consistent after various statistical adjustments [139]. However, significant heterogeneity was observed in patients with in-stent restenosis, where OCT-guided PCI was superior to IVUS-guided PCI. Additionally, there was a significant reduction of approximately 1.5% in target-vessel myocardial infarctions with OCT-guided PCI [139]. While results from secondary analyses should be considered as hypothesis-generating and not overemphasized, this outcome may be linked to variations in lesion preparation and stent sizing strategies between the two guidance methods [140]. Taken together, these data suggest that specific lesion subsets may derive differential benefit from IVI in general (e.g., left main, ostial, long diffuse, and chronic total occlusion lesions as studied in RENOVATE-COMPLEX-PCI) and from OCT guidance in particular (e.g., bifurcations in OCTOBER and restenotic lesions in OCTIVUS) [127,140,141].

### 4.4. Critical Reading of Current Evidence

A critical evaluation of the accumulated evidence confirms that IVI guidance yields superior outcomes compared with angiographic guidance. The RENOVATE-Complex PCI trial, which randomized 1639 patients with at least one prespecified complex coronary lesion to imaging-guided (e.g., IVUS or OCT at the operator’s discretion) versus angiography-guided PCI, exemplifies the incremental value of IVI in contemporary clinical practice [141]. At a median follow-up of 25 months, the primary composite endpoint of cardiac death, target-vessel myocardial infarction, and clinically driven target-vessel revascularization occurred in 7.7% of patients in the IVI-guided group and in 12.3% of patients in the angiography group (HR 0.64; 95% CI 0.45 to 0.89; *p* = 0.008) for a significant absolute risk reduction of 4.6% [141]. This benefit persisted after adjustment for baseline anatomical complexity and was consistent across lesion subsets [141]. These findings are further supported by a comprehensive meta-analysis, which pooled data from 24 randomized trials [17]. In a pairwise comparison framework, both frequentist and Bayesian analyses demonstrated that IVI-guided PCI was associated with a significant reduction in target lesion revascularization, cardiac death and stent thrombosis [17]. While the precise mechanisms underlying the mortality benefit remain multifactorial and partially unclear, a plausible contributor is the observed reduction in definite or probable stent thrombosis, a recognized cause of sudden cardiac death [17]. However, sensitivity analyses indicated that the strength and robustness of the stent thrombosis signal varied across statistical approaches and were disproportionately influenced by a small number of influential trials [17].

On the other hand, important nuances emerge when the individual IVI modalities are evaluated against each other. While direct comparisons between IVUS and OCT for clinical outcomes are limited, essentially relying on the OPINION and OCTIVUS trials, evidence from a network meta-analysis may offer additional perspectives. In a frequentist and Bayesian network meta-analysis IVUS, but not OCT, was associated with a significant reduction in all-cause death compared to angiographic guidance, whereas neither IVI modality demonstrated a significant reduction in myocardial infarction [17,134,135]. The combination of the evidence coming from trials testing OCT with that of trials testing IVUS in a pairwise meta-analysis was required to observe a benefit in myocardial infarction compared to angiography alone [17]. Inconsistencies emerged across several endpoints, particularly target-lesion revascularization and MACE, largely due to the influence of the neutral ILUMIEN IV trial, which carried substantial statistical weight and disrupted the assumption of transitivity [17]. Specifically, if IVUS is superior to angiography and OCT is comparable to IVUS, then OCT would be expected to outperform angiography, a relationship not upheld once ILUMIEN IV was incorporated in the model [17]. In a similar meta-analysis by Stone and colleagues, the comparison between IVUS and OCT, based on five randomized trials, yielded consistent findings, showing comparable performance between the two modalities in terms of target-lesion failure and the other outcomes examined [109].

Taken together, contemporary evidence supports IVI as the reference standard for guiding and optimizing stent deployment, primarily through consistent reductions in repeat revascularization and potential benefits in lowering cardiac death and stent thrombosis. Nevertheless, important uncertainties remain, particularly in delineating the specific anatomical and clinical contexts in which each modality confers the greatest procedural and prognostic advantages and should be systematically used for guiding PCI. It should also be noted that much of the randomized evidence supporting IVI-guided PCI, particularly IVUS-guided stent optimization, has been generated in East Asian populations, where IVI is routinely employed and reimbursement is more favorable. By contrast, OCT and IVUS adoption remain more heterogeneous across Europe and North America. As a result, reported gains in procedural success and clinical outcomes with IVI-guided PCI may not be directly generalizable to all healthcare systems, ethnic backgrounds, or reimbursement environments, and should be interpreted in the context of regional practice patterns. Future randomized trials focused on high-risk lesion subsets, alongside individual-patient-level meta-analyses, are essential to clarify these differential effects, enhance cost-effectiveness assessments, and support tailored imaging selection in routine clinical practice.

## 5. Future Perspectives

OCT and IVUS provide complementary information by overcoming the limitations of each technique and enhancing anatomical and procedural insights. However, such sequential imaging is rarely applicable in daily practice, and its clinical utility remains underexplored and likely underestimated. This approach requires time, experience, and technical skills to match corresponding cross-sections (co-registration) and to align image orientations (co-alignment). Moreover, it introduces additional procedural time, cost, potential risk of complications, and often suffers from imprecise co-registration and image fusion. A hybrid IVUS-OCT catheter has the potential to address these limitations. By combining IVUS and OCT in a single platform, hybrid systems promise to streamline workflow, reduce contrast load, and expand the clinical utility of IVI [142]. Advanced platforms that enable real-time OCT-IVUS co-registration and display overlapped or side-by-side images, such as the Conavi Novasight Hybrid, Dual-Sensor, and Panovision S1 systems, are now in late-feasibility phases and have entered early clinical evaluation [143,144]. Preliminary findings with these systems have shown that simultaneous image acquisition and postprocessing are technically feasible without compromising catheter profile or crossability, and without prolonging procedural times and increasing complications compared with individual IVI modalities.

While current IVI modalities are primarily used for procedural guidance and lesion characterization, the field is also evolving toward molecular imaging and image-guided interventions, a shift that may be particularly relevant in the management of non-culprit lesions [14]. Emerging technologies such as intravascular photoacoustic imaging (IVPA), which enables detection of lipid accumulation, inflammatory cell infiltration, and endothelial dysfunction, and near-infrared autofluorescence (NIRAF), which identifies signals associated with intraplaque hemorrhage and oxidative stress, expand the diagnostic potential of IVI beyond structural assessment to molecular and metabolic characterization of atherosclerosis [142].

In this context, near-infrared fluorescence (NIRF) molecular imaging, requiring systemic administration of activatable fluorophores targeted to specific inflammatory or metabolic plaque components, introduces a promising therapeutic dimension [142]. Once activated by external or catheter-delivered near-infrared light, these tracers emit fluorescence signals that are detectable by specialized catheters. Proof-of-concept animal studies have demonstrated that this strategy not only allows for the identification of inflamed plaques but also enables localized phototherapy. Specifically, photoactivatable agents targeted to macrophages can be activated intravascularly to suppress inflammation and promote a shift toward fibrotic plaque remodeling [142,145]. This integrated diagnostic-therapeutic approach effectively bridges the gap between diagnosis and therapy, positioning IVI as a future platform for molecularly guided, lesion-specific intervention [142].

Table 5 provides an overview of ongoing randomized trials investigating IVI in PCI. As these technologies evolve, the evidence base will grow through ongoing randomized trials, which continue to compare IVUS or OCT with angiography in selected high-risk populations, such as those with complex multivessel disease, unprotected left main, bifurcation lesions, chronic kidney disease, ST-segment–elevation myocardial infarction, or in-stent restenosis. To date, no ongoing trial is comparing IVUS and OCT head-to-head, leaving the relative merits of each modality an unresolved question.

Despite guideline recommendations, use of IVI in PCI remains inconsistent and, in some healthcare systems, clearly suboptimal, likely due to structural barriers (e.g., reimbursement, resource allocation). Together with scientific progress, the achievement of broader IVI adoption will require dedicated reimbursement strategies that recognize IVI-guided PCI as a quality standard rather than an optional add-on, structured operator training to counter the perception that IVI adds time and complexity, and seamless integration of IVI-derived measurements into workflow, ultimately establishing IVI as a marker of procedural quality and patient prognosis.

Together, these advances suggest that the role of IVI will continue to evolve from procedural planning and optimization toward a growing role in guiding therapeutic strategies for non–flow-limiting plaques that are prone to rupture [32,33,34,35].

## 6. Conclusions

IVI stands out as the reference standard for guiding PCI, offering procedural and clinical advantages over angiographic guidance particularly in anatomically complex coronary lesions. The evidence supporting IVI is grounded in randomized trials and meta-analyses. Nevertheless, the significant heterogeneity in the magnitude and strength of short- and long-term improvements in clinical outcomes across the available studies raises questions about the settings in which IVI should be systematically used, and one modality confers the greatest incremental benefit over the others. Head-to-head trials have not demonstrated clear clinical superiority of one IVI modality over the others, and current data suggest that optimal modality selection should be individualized according to lesion characteristics, operator’s expertise, and resource availability. Future studies are needed to refine imaging thresholds, validate lesion-specific strategies, and determine the comparative cost-effectiveness between IVI modalities.

## Figures and Tables

**Table 1:** Recommendations from American and European guidelines on the use of intravascular imaging during percutaneous coronary intervention.

Clinical Setting	Society	Year	Technique	Subset	Class	LOE
**Acute Coronary Syndromes**	ACC/AHA/SCAI	2025	IVUS or OCT	PCI in left main artery or in complex lesions	I	A
	ESC/EAPCI	2023	IVUS or OCT	PCI	IIa	A
	ESC/EAPCI	2023	OCT	Ambiguous culprit lesions	IIb	C
**Chronic Coronary Syndromes**	ACC/AHA	2023	-	-	-	-
	ESC/EAPCI	2024	IVUS or OCT	Complex PCI (left main, true bifurcations, long lesions)	I	A
**Myocardial Revascularization**	ESC/EACTS	2018	IVUS	Unprotected left main revascularization	IIa	B
	ESC/EACTS	2018	IVUS and/or OCT	Detection of ISR causes	IIa	C
	ESC/EACTS	2018	IVUS or OCT	Optimize stent implantation in selected patients	IIa	B
	ACC/AHA/SCAI	2021	IVUS	Intermediate left main stenosis	IIa	B
	ACC/AHA/SCAI	2021	IVUS	PCI of left main or complex lesions	IIa	B
	ACC/AHA/SCAI	2021	OCT	Complex lesions except ostial left main	IIa	B
	ACC/AHA/SCAI	2021	IVUS or OCT	Detection of stent failure	IIa	C

Abbreviations: ACC, American College of Cardiology; AHA, American Heart Association; SCAI, Society for Cardiovascular Angiography and Interventions; ESC, European Society of Cardiology; EAPCI, European Association of Percutaneous Cardiovascular Interventions; EACTS, European Association for Cardio-Thoracic Surgery; ISR, in-stent restenosis; IVUS, Intravascular Ultrasound; OCT, Optical Coherence Tomography; PCI, Percutaneous Coronary Intervention; LOE, Level of Evidence.

**Table 2:** Randomized trials assessing the use of intravascular ultrasound versus invasive coronary angiography.

Trial	Sample Size	Clinical Presentation (%)	Diabetes (%)	Multivessel Disease (%)	Bifurcations (%)	Left Main (%)	Primary Endpoint	Maximum Follow-Up	Objective Met
**AIR-CTO [112]**	230	CCS: 73.5 UA: 9.2 AMI: 24	28.3	83.9	14.8	-	In-stent LLL	24	Yes
**AVIO [113]**	284	UA: 27.9	25.4	-	19.1	0	Post-PCI in-lesion MLD	24	Yes
**CTO-IVUS [119]**	402	CCS: 100.0	34.3	67.2	24.9	0	Cardiac death	12	No
**DOCTORS [92]**	240	UA: 19.0 ACS: 81.0	18.8	30.8	-	0	Post-PCI FFR	6	Yes
**GUIDE-DES [117]**	1528	CCS: 70.9 NSTE-ACS: 22.0 STEMI: 7.1	32.3	50	67.2	12.8	Cardiac death, TVMI or ID-TLR	12	Yes
**HOME DES IVUS [120]**	210	CCS: 39.0 UA/NSTE-ACS: 41.0 STEMI: 25.0	43.5	57	-	3.5	Death, myocardial infarction, and TLR	18	No
**IVUS-XPL [114]**	1400	CCS: 61.4 ACS: 38.6	31.7	-	0	0	Cardiac death, TLMI, or ID-TLR	60	Yes
**Li et al. [121]**	228	CCS: 100.0	100.0	53.9	36.8	17.0	Cardiac death, nonfatal MI, and TLR	24	Yes
**Liu et al. [122]**	336	CCS: 13.4 UA: 75.3 Recent MI: 11.3	32.1	83.6	60.1	100.0	Cardiac death, MI, TLR. Stent thrombosis	12	Yes
**RESET [123]**	543	CCS: 52.3 UA: 38.3 AMI: 9.4	30.8	39.0	0	0	Cardiac death, MI, TVR, or stent thrombosis	12	No
**Tan et al. [124]**	123	Stable Angina: 31.8 UA: 68.2	31.7	86.2	53.7	100	Death, non-fatal MI, or TLR	24	Yes
**ULTIMATE [87]**	1448	CCS: 21.5 UA: 65.8 AMI: 12.5	30.6	54.9	25.0	9.2	Cardiac death, TVMI, and clinically driven TVR	36	Yes
**Wang et al. [125]**	80	STEMI: 100	16.3	-	7.5	0	Cardiac death, MI, target vascular reconstruction, and intractable myocardial ischemia	12	No
**IVUS-ACS [118]**	3505	ACS: 100	31.5	7.0	15.2	4.1	Cardiac death, TVMI, TVR	12	Yes

Abbreviations: ACS, Acute Coronary Syndrome; AMI, Acute Myocardial Infarction; CCS, Chronic Coronary Syndrome; FFR, Fractional Flow Reserve; ID-TLR, Ischemia-Driven Target Lesion Revascularization; IVUS, Intravascular Ultrasound; MI, Myocardial Infarction; MLD, Minimal Lumen Diameter; NSTE-ACS, Non-ST-Elevation Acute Coronary Syndrome; OCT, Optical Coherence Tomography; PCI, Percutaneous Coronary Intervention; STEMI, ST-Elevation Myocardial Infarction; TLMI, Target Lesion-Related Myocardial Infarction; TLR, Target Lesion Revascularization; TVMI, Target Vessel Myocardial Infarction; TVR, Target Vessel Revascularization; UA, Unstable Angina.

**Table 3:** Randomized trials assessing the use of optical coherence tomography versus invasive coronary angiography.

Trial	Sample Size	Clinical Presentation (%)	Diabetes (%)	Multivessel Disease (%)	Bifurcations (%)	Left Main (%)	Primary Endpoint	Maximum Follow-Up	Objective Met
**ILUMIEN IV [90]**	2487	CCS: 42.5 UA: 27.6 ACS: 29.9 Staged PCI: 5.7	42.0	-	3.3	0	Post-PCI MSA. Cardiac death, TVMI, or ID-TVR	24	No
**Kala et al. [129]**	201	CCS: 51.0 UA: 33.5 AMI: 15.5	36.5	68.6	0	0	Death, myocardial infarction, and TLR	9	No
**Kim et al. [130]**	101	STEMI: 100.0	21.3	10.6	-	0	Percentage of uncovered struts	12	Yes
**OCTACS [131]**	100	NSTE-ACS: 100.0	13	38.0	0	0	Percentage of uncovered struts	6	Yes
**EROSION III [132]**	246	ACS: 100.0	21.2	-	-	0	Rate of stent implantation	12	Yes
**OCTOBER [127]**	1201	CCS: 54.2 UA: 9.2 NSTE-ACS: 13.1 Staged PCI: 23.5	16.7	18.9	100.0	16.5	Cardiac death, TLMI, or ID-TLR	24	Yes
**CALIPSO [126]**	143	CCS: 100	38.0	-	-	7.0	Post-PCI MSA	12	Yes
**OCCUPI [128]**	1604	Stable: 52 NSTEMI: 14 STEMI: 7	33.0	-	24.0	14.0	Cardiac death, MI, stent thrombosis, or ID-TLR	12	Yes

Abbreviations: ACS, Acute Coronary Syndrome; AMI, Acute Myocardial Infarction; CCS, Chronic Coronary Syndrome; ID-TLR, Ischemia-Driven Target Lesion Revascularization; ID-TVR, Ischemia-Driven Target Vessel Revascularization; MI, Myocardial Infarction; MSA, Minimal Stent Area; NSTE-ACS, Non-ST-Elevation Acute Coronary Syndrome; PCI, Percutaneous Coronary Intervention; STEMI, ST-Elevation Myocardial Infarction; TLMI, Target Lesion Myocardial Infarction; TLR, Target Lesion Revascularization; TVMI, Target Vessel Myocardial Infarction; TVR, Target Vessel Revascularization; UA, Unstable Angina.

**Table 4:** Randomized trials assessing the use of intravascular ultrasound versus optical coherence tomography.

Trial	Sample Size	Clinical Presentation (%)	Diabetes (%)	Multivessel Disease (%)	Bifurcation (%)	Left Main (%)	Primary Endpoint	Maximum Follow-Up (Months)	Objective Met
**ILUMIEN III [84]**	450	CCS: 63.5 UA: 18.9 NSTE-ACS: 14 STEMI: 3.6	33.1	-	0	0	Post-PCI MSA.	12	Yes
**iSIGHT [137]**	151	CCS: 40.6 UA/NSTE-ACS: 38.7 Recent MI: 20.7	39.3	-	0	0	Post-PCI stent expansion	30	Yes
**MISTIC-1 [138]**	109	CCS: 100.0	46.8	40.3	-	0	In-segment MLA	36	Yes
**OPINION [135]**	829	CCS: 87.5 UA: 12.5	40.9	-	38.4	0	Cardiac death, TVMI, and ID-TVR	12	Yes
**OCTIVUS [134]**	2008	CCS: 76.6 UA: 13.5 NSTE-ACS: 9.9	33.3	61.6	52.6	13.5	Cardiac death, TVMI, ID-TVR	24	Yes
**OPINION-ACS [133]**	158	STEMI: 55 NSTEMI: 29 UA: 16	35	-	-	-	In-stent MLA	8	Yes

Abbreviations: CCS, Chronic Coronary Syndrome; ID-TVR, Ischemia-Driven Target Vessel Revascularization; IVUS, Intravascular Ultrasound; MLA, Minimum Luminal Area; MSA, Minimum Stent Area; NSTE-ACS, Non-ST-Elevation Acute Coronary Syndrome; NSTEMI, Non-ST-Elevation Acute Myocardial Infarction; PCI, Percutaneous Coronary Intervention; STEMI, ST-Elevation Myocardial Infarction; TVMI, Target Vessel Myocardial Infarction; UA, Unstable Angina.

**Table 5:** Ongoing randomized trials assessing the use of intravascular imaging in percutaneous coronary intervention.

Trial Name, Registration Number	Intervention	Control	Sample Size	Key Population Characteristics	Primary Endpoint	Estimated Study End (Year)
**IVUS-CHIP,** NCT04854070	IVUS-guided PCI	Angiography-guided PCI	2020	Complex/high-risk lesions: heavy calcification, bifurcation, left main, CTO, long lesions, ISR	Target-vessel failure at 12 months	2027
**IMPROVE,** NCT04221815	IVUS-guided PCI	Angiography-guided PCI	3100	Complex lesions (CTO, ISR, calcification, long ≥28 mm, bifurcation)	Target-vessel failure at 12 months	2026
**OPTIMAL,** NCT04111770	IVUS-guided PCI	Angiography-guided PCI	800	Unprotected left-main CAD (ostial, shaft, distal bifurcation)	Death, stroke, MI, or clinically indicated revascularization at 24 months	2025
**DKCRUSH VIII,** NCT03770650	IVUS-guided DK-Crush stenting	Angiography-guided DK-Crush	556	True bifurcation lesions (DEFINITION criteria)	Cardiac death, TV-MI, or TVR at 12 months	2025
**IVUS-CKD,** NCT06567938	IVUS-guided PCI	Angiography-guided PCI	1528	Chronic kidney disease (eGFR <60 mL min^−1^ 1.73 m^−2^)	Target-vessel failure at 12 months	2027
**INSIDE-OCT,** NCT06779110	OCT-guided PCI	Angiography-guided PCI	360	In-stent restenosis 70–99%, vessel 2.25–5.75 mm; stable CAD or ACS	Post-PCI minimal-stent area (core lab)	2028
**FRAME-AMI3,** NCT06227754	OCT-guided PCI	Angiography-guided PCI	1500	Patient with STEMI	Target Vessel Failure 24 months	2028

Abbreviations: ACS, Acute Coronary Syndrome; CAD, Coronary Artery Disease; CTO, Chronic Total Occlusion; DK, Double Kissing; eGFR, Estimated Glomerular Filtration Rate; ISR, In-Stent Restenosis; IVUS, Intravascular Ultrasound; MI, Myocardial Infarction; OCT, Optical Coherence Tomography; PCI, Percutaneous Coronary Intervention; STEMI, ST-Elevation Myocardial Infarction; TV-MI, Target Vessel Myocardial Infarction; TVR, Target Vessel Revascularization.

## Data Availability

Not applicable.
